# Removal of soil biota alters soil feedback effects on plant growth and defense chemistry

**DOI:** 10.1111/nph.15485

**Published:** 2018-10-17

**Authors:** Minggang Wang, Weibin Ruan, Olga Kostenko, Sabrina Carvalho, S. Emilia Hannula, Patrick P. J. Mulder, Fengjiao Bu, Wim H. van der Putten, T. Martijn Bezemer

**Affiliations:** ^1^ College of Life Sciences Nankai University Tianjin 300071 China; ^2^ Department of Terrestrial Ecology Netherlands Institute of Ecology (NIOO‐KNAW) PO Box 50 6700 AB Wageningen the Netherlands; ^3^ Department of Plant Protection Biology Swedish University of Agricultural Sciences PO Box 102 SE‐23053 Alnarp Sweden; ^4^ RIKILT – Wageningen University & Research PO Box 230 6700 AE Wageningen the Netherlands; ^5^ Laboratory of Molecular Biology Department of Plant Sciences Wageningen University & Research Wageningen the Netherlands; ^6^ Laboratory of Nematology Wageningen University & Research PO Box 8123 6700 ES Wageningen the Netherlands; ^7^ Institute of Biology Section Plant Ecology and Phytochemistry Leiden University PO Box 9505 2300 RA Leiden the Netherlands

**Keywords:** fractionation, *Jacobaea vulgaris*, plant–soil feedback (PSF), pyrrolizidine alkaloids (PAs), soil biota, spectral reflectance

## Abstract

- We examined how the removal of soil biota affects plant–soil feedback (PSF) and defense chemistry of *Jacobaea vulgaris*, an outbreak plant species in Europe containing the defense compounds pyrrolizidine alkaloids (PAs).
- Macrofauna and mesofauna, as well as fungi and bacteria, were removed size selectively from unplanted soil or soil planted with *J. vulgaris* exposed or not to above‐ or belowground insect herbivores. Wet‐sieved fractions, using 1000‐, 20‐, 5‐ and 0.2‐μm mesh sizes, were added to sterilized soil and new plants were grown. Sieving treatments were verified by molecular analysis of the inocula.
- In the feedback phase, plant biomass was lowest in soils with 1000‐ and 20‐μm inocula, and soils conditioned with plants gave more negative feedback than without plants. Remarkably, part of this negative PSF effect remained present in the 0.2‐μm inoculum where no bacteria were present. PA concentration and composition of plants with 1000‐ or 20‐μm inocula differed from those with 5‐ or 0.2‐μm inocula, but only if soils had been conditioned by undamaged plants or plants damaged by aboveground herbivores. These effects correlated with leaf hyperspectral reflectance.
- We conclude that size‐selective removal of soil biota altered PSFs, but that these PSFs were also influenced by herbivory during the conditioning phase.

## Introduction

A rapidly increasing number of studies are revealing how plant–soil feedbacks (PSFs) influence individual plant performance and plant population dynamics (Johnson *et al*., 2012; Revilla *et al*., 2013; Maron *et al*., 2014). The majority of these studies report negative conspecific PSF effects, indicating that many plants grow poorer in soil previously colonized by individuals of the same species than in sterilized soil or in soil conditioned by other plant species (Kulmatiski *et al*., 2008). Negative PSF can be caused by plant‐mediated changes in the composition of the soil biotic community that influences the growth of plants following up in that soil (Bever *et al*., 1997; van der Putten *et al*., 2013). So far, PSF studies have mainly focused on the net effects on plant performance of whole soil assemblages using a ‘black box’ approach. Therefore, it is still poorly understood which specific groups of soil organisms contribute to PSF effects (van de Voorde *et al*., 2012; Bezemer *et al*., 2013).

Soil organisms differ in size and can be separated to a large extent using wet‐sieving approaches (Bradford *et al*., 2002; Wagg *et al*., 2014). Hence, sieving out size fractions from the soil community can be used to disentangle the effects of different‐sized soil organisms on PSF. For example, inoculation of microfauna using a 40‐μm mesh extracted from soil in which *Jacobaea vulgaris* plants had been grown resulted in weaker negative conspecific PSF effects than inoculation of both micro‐ and mesofauna (1000‐μm mesh) (van de Voorde *et al*., 2012). Within the microfauna community, soil organisms, such as nematodes, fungi and bacteria, may also, at least partly, be separated by sieving through different‐sized meshes. In a microcosm study, re‐inoculation of separately sieved fractions to sterilized soil altered plant–soil interactions and ecosystem functioning (Wagg *et al*., 2014). In the present study, we used the plant species *J. vulgaris*, which shows a strong negative conspecific feedback (van de Voorde *et al*., 2011), to examine how the inoculation of different‐sized groups of soil organisms obtained by sieving influences PSF effects.

PSF studies typically focus on changes in plant biomass (Klironomos, 2002; Kulmatiski *et al*., 2008; Brinkman *et al*., 2010). However, the interactions between a plant and its associated soil organisms can also influence the concentration or composition of secondary metabolites of that plant, which may play a role in the defense of plants against their natural enemies (Erb *et al*., 2009; Mithoefer & Boland, 2012). Plant defense responses induced by soil organisms can occur locally in infested or damaged plant tissues, as well as systemically in other parts of the plant (Bardgett & Wardle, 2010; van Dam & Heil, 2011). Recent studies have shown that plant‐induced changes in the composition of the soil microbial community can even influence the concentration of pyrrolizidine alkaloids (PAs) in the foliage of *J. vulgaris* plants which grow subsequently in that soil (Bezemer *et al*., 2013; Kos *et al*., 2015). Remarkably, these soil feedback effects on PAs can also be influenced by foliar‐feeding or root‐feeding herbivores which have been feeding on the preceding plants (Kostenko *et al*., 2012; Bezemer *et al*., 2013). Therefore, an open question is whether and how different‐sized groups of soil organisms influence PSF‐induced plant defense responses and how this interacts with herbivory during the conditioning phase.

Recent studies have assessed plant responses to environmental stresses by measuring the spectral reflectance patterns of the plant (Ferwerda *et al*., 2005; Skidmore *et al*., 2010; Asner & Martin, 2011). This is because hyperspectral reflectance of specific narrow spectral bands in plant tissues can reflect subtle features that link to specific plant chemical compounds (Curran *et al*., 1992; Fourty *et al*., 1996). Carvalho *et al*. (2014) showed that the concentration and composition of PAs in the foliage of *J. vulgaris* could be linked to specific spectral reflectance patterns in the leaves of this species. Here, one aspect of study is whether changes in soil communities can be detected via the measurement of the hyperspectral reflectance patterns of plants growing in soils with different soil communities (Carvalho *et al*., 2012).

In the present study, we selectively removed groups of soil organisms based on size by filtering soil suspensions obtained from unconditioned soil collected from the field and from soil conditioned by *J. vulgaris*. These soils were filtered through a series of filters ranging from 1000‐ to 0.2‐μm mesh. During the conditioning phase, additional treatments were included by exposing plants to aboveground (AG) or belowground (BG) insect herbivory. We determined the presence and composition of the bacterial and fungal communities in the different filtered suspensions, and measured the biomass, the concentration and composition of PAs and the hyperspectral reflectance patterns of *J. vulgaris* plants growing in sterile soil inoculated with these suspensions (named soil inocula). We tested the following hypotheses: (1) soil conditioning by *J. vulgaris* plants will result in negative PSF and in increased concentrations, as well as in altered composition, of PAs in the foliage of succeeding plants, (2) the magnitude of PSF on plant growth and PAs will be enhanced when, during the conditioning phase, plants are exposed to AG and BG herbivory, (3) PSF effects on plant growth and PAs will be reduced by the progressive exclusion of soil organisms, and (4) the changes in plant growth and PAs in differently inoculated soils will correlate with the hyperspectral reflectance patterns of these plants.

## Materials and Methods

In this study, we used ragwort, *Jacobaea vulgaris* (synonym *Senecio jacobaea* L., Asteraceae), as a study system. *Jacobaea vulgaris* is native to Europe, but is highly invasive in other continents (e.g. Poole & Cairns, 1940; Harper & Wood, 1957; Harris *et al*., 1971). In Europe, this species typically dominates in former arable fields, but often declines over time because of a negative intraspecific PSF which may be caused by the accumulation of soil pathogens (Bezemer *et al*., 2006; van de Voorde *et al*., 2011; Wubs & Bezemer, 2016). This plant species contains PAs that are toxic to various herbivores (Johnson *et al*., 1985; Gardner *et al*., 2006; Liu *et al*., 2017). PAs in *J. vulgaris* are root‐produced secondary metabolites (Hartmann, 1999) and are generally contained in plant tissues in the tertiary amine (free base) form and in the N‐oxide form. Tertiary amines are regarded as degradation products of N‐oxides (Hartmann & Dierich, 1998) and are more toxic for herbivorous insects than their corresponding N‐oxides (van Dam *et al*., 1995; Macel *et al*., 2005).

### Seed and soil collection

Seeds of *J. vulgaris* were collected from a single adult plant growing in a restoration grassland on former arable land at Mossel, Veluwe, the Netherlands (52°04′N, 05°44′E) where agricultural practices were ceased in 1995. Seeds were sterilized with sodium hypochlorite (2%) for 1 min, rinsed four times with demi‐water and germinated on oven‐sterilized (at 110°C) glass beads in plastic trays that had been surface sterilized using 70% ethanol. The germination trays were placed in an artificially illuminated growth chamber at 22°C. Ten‐day‐old seedlings were used in phase I of the experiment (see ‘Experimental setup’ below for phase I description). The remaining seedlings were kept in an illuminated growth chamber at 4°C for use in phase II.

Soil was collected at 0–15‐cm depth below the soil surface from the same location from which seeds were collected. The soil was a sandy loam with a particle size distribution of 3% at < 2 μm, 17% at 2–63 μm, 80% at > 63 μm and 4.5% organic matter content. After collection, soil was immediately sieved through a 5‐mm mesh and thoroughly mixed. The sieved soil was divided into two parts: live soil that was used in phase I of the experiment, and the remaining soil that was sterilized by gamma irradiation (> 25 kGy) at Isotron (Ede, the Netherlands) for phase II.

### Experimental setup

**Figure 1:**
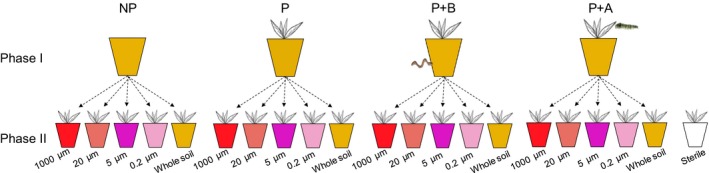
Scheme of the experimental design. Phase I (conditioning phase): field soil was kept in the glasshouse in pots without a plant (not conditioned, NP), in pots with a *Jacobaea vulgaris* plant (P), and in pots with a *J. vulgaris* plant exposed to belowground herbivory (P+B) or aboveground herbivory (P+A). Phase II (feedback phase): soil inocula obtained from soil in phase I were added to pots filled with sterilized soil. Soil inocula were either obtained from soil suspensions that passed through filters of different mesh size (1000, 20, 5 and 0.2 μm) or from whole live soil (named ‘Whole soil’ and mixed with sterilized soil at 1 : 1 w/w). ‘Sterile’ treatment consisted of sterilized soil to which the same volume of demineralized water was added.

**Phase I (conditioning phase).** We used live field soil to create four treatments (see Fig. 1): (1) soil with no plants (abbreviated as ‘NP’), (2) soil conditioned by *J. vulgaris* plants (abbreviated as ‘P’), (3) soil conditioned by *J. vulgaris* exposed to BG herbivory (abbreviated as ‘P+B’) and (4) soil conditioned by *J. vulgaris* exposed to AG herbivory (abbreviated as ‘P+A’). Forty surface‐sterilized pots were prepared using 70% ethanol for each treatment. Each pot (13 × 13 × 13 cm^3^) was filled with 1.6 kg of field soil and the soil surface was covered with 100 g of coarse white sand to prevent oviposition by fungus gnats. For all plant conditioning treatments (P, P+B and P+A), one individual *J. vulgaris* seedling was transplanted into each pot. All pots were immediately watered and randomly placed on trolleys in a glasshouse with a 21°C : 16°C temperature regime and a 16 h : 8 h, day : night photoperiod. Natural daylight was supplemented by 400 W metal halide lamps (225 μmol m^−2^ s^−1^ photosynthetically active radiation, PAR).

Six weeks after transplantation, three wireworm individuals (*Agriotes lineatus*) were placed into 1‐cm‐deep holes made in the soil to initiate the ‘P+B’ treatment. After the larvae had burrowed into the holes, the holes were immediately covered by surface soil in the pot. All wireworms were maintained in pots for 8 wk until harvest. Similar holes were made in the soil of all other pots. The ‘P+A’ treatment was created by introducing one third‐instar *Mamestra brassicae* larva in a clip‐cage (2 cm in diameter) on the youngest fully mature leaf, so that an area of 3.14 cm^2^ was consumed over a period of 1–2 d. This was performed five times over 8 wk until harvest. Empty clip‐cages were placed on identically aged leaves of plants in the other treatments.

All pots were watered three times per week and reweighed to adjust the soil moisture level to 17% once a week. The position of the trolleys was rotated every week to avoid position effects. All pots were fertilized five times with a total of 120 ml of half‐strength Hoagland nutrient solution and were harvested 14 wk after transplantation. At harvest, plant shoots (P, P+B, P+A treatments) were clipped at the soil surface and roots were carefully removed from the soil. Soil from all pots was then used to prepare soil inocula for phase II. As the preparation of the soil inocula was a time‐consuming process, the pots were harvested in blocks. Each block contained pots from all treatments.

Four types of watery soil inocula were prepared for each conditioning treatment (Fig. 1). Each inoculum originated from a separate pot to avoid pseudoreplication.

1. 1000‐μm inoculum: 720 g of demineralized water was added to a homogenized sample of 800 g of soil collected from a single pot (water : soil = 90 : 100, w/w) in a 2‐l beaker. This procedure was chosen because preliminary trials revealed that *c*. 120 ml of suspension can be obtained from 225 ml of demineralized water suspended with 250 g of 17% moisture soil (water : soil = 90 : 100, w/w). The soil suspension obtained was stirred for 30 s and, after letting the soil particles settle for 30 s, the suspension was sieved through a sterilized 1000‐μm pore‐size sieve. The filtrate obtained was collected in a new plastic container.
2. 20‐μm inoculum: the procedure described for (1) was followed to obtain a soil suspension. This soil suspension was then gently filtered through a 20‐μm pore‐size sieve and the filtrate was collected in a new container.
3. 5‐μm inoculum: the procedure described for (1) was followed. The suspension was left to rest for 4 h until the sediment had settled and then transferred to a new container using a 50‐ml syringe connected to a flexible plastic tube. The suspension obtained was consecutively filtered through 8‐μm filter paper (Whatmann; diameter, 150 mm) and a 5‐μm nitrate cellulose membrane (Whatmann) placed inside a syringe and finally collected in a new container. The syringe was thoroughly rinsed with 0.2‐μm‐filtered deionized water (Millipore) after usage, and each membrane was used only once.
4. 0.2‐μm inoculum: the procedure described in (1) was followed. The suspension was left to rest for 4 h so that the sediment had settled. The settled suspension was consecutively filtered through an 11‐μm filter paper (Whatmann: diameter, 150 mm), a 3‐μm filter paper and a 0.2‐μm nitrate cellulose membrane syringe filter (Whatmann), and collected in a new container.

Via the abovementioned filtration steps, soil biota were largely separated into different groups. Soil biota with sizes larger than 1000 μm were excluded in this study. Most soil mesofauna, such as microarthropods, nematodes, enchytraeids and collembola, were excluded via filtration through a 20‐μm pore mesh (van de Voorde *et al*., 2012). Most soil fungi were excluded via filtration through a 5‐μm pore mesh, although some yeasts or fungal spores may, in theory, pass through this sieving level (Duarte *et al*., 2008); microbiota, including most bacteria, were excluded when filtering through a 0.2‐μm pore mesh. Although the different mesh sizes were assumed to exclude different groups of soil biota, they may also have excluded different‐sized soil particles when a proportion of soil biota had settled over the filtrations, and this could also potentially contribute to differences in microbial composition amongst the inocula.

In addition, we also mixed live soil (hereafter named ‘whole soil’ inoculum to distinguish from watery inocula) with sterilized soil (1 : 1, w/w) to examine overall PSFs under various soil conditioning treatments. Clearly, whole soil inoculum may have soil communities that differ from those of the filtered watery inocula, and also when compared with the 1000‐μm watery inoculum which is supposed to contain the most complex soil community. These differences between whole soil inoculum and watery soil inocula can lead to different plant responses in the feedback phase, and hence the two types of treatment are not directly comparable.

For each watery inocula treatment, 120 ml of inoculum was added to a pot (9 × 9 × 10 cm^3^) filled with 500 g of previously sterilized field soil. Each pot was placed on a plastic dish (15 cm in diameter) to ensure that potential leachate was reabsorbed and to prevent contamination between pots. There were 10 replicates for the 1000‐, 20‐ and 5‐μm watery inocula treatments, but seven replicates for the 0.2‐μm inoculum treatment because the preparation of this inoculum was very time consuming. Subsamples of all watery inocula (1000, 20, 5 and 0.2 μm) and of the whole soil inoculum for each replicate were collected into 2‐ml Eppendorf tubes and immediately stored at −80°C until further use for molecular analysis. Ten extra pots filled with 500 g of sterilized soil and 120 ml of double‐distilled demineralized water were included as a control. In total, there were 158 pots ((10 + 10 + 10 + 7 replicates) × 4 treatments + 10 sterile control). After inoculation, all pots were immediately covered with aluminum foil until seedling transplantation to protect the soil from desiccation.

#### Phase II (feedback phase)

One week after soil inoculation had been completed, one *J. vulgaris* seedling was transplanted into each pot. The plant growth conditions were identical to the conditions in the conditioning phase. Five weeks after transplantation, the leaf spectral reflectance of the first fully expanded leaf of each plant was measured. The leaf was then immediately removed from the plant using a razor blade. The detached leaf was scanned (Perfection 4990; Epson, Nagano, Japan) to determine the surface area using Winfolia pro 2006 (Regent Instruments Inc., Quebec, Canada), and then freeze‐dried under vacuum (3 d at a collector temperature of −55°C; Labconco Free Zone 12 L Freeze Dry System, Kansas City, MO, USA) for the determination of dry weight and later chemical analysis. All plants were harvested 1 d after leaf spectral measurements. At harvest, AG plant material was clipped and roots were carefully rinsed from the soil. Shoot and root biomass was dried at 70°C for 5 d and the dry weight was determined.

### Leaf spectral measurements

A plant probe with leaf‐clip attached to an ASD Fieldspec‐3 field spectrometer (ASD Inc., Boulder, CO, USA) was used to determine spectral reflectance. The light bulb used in the probe was a heat‐sensitive halogen lamp (temperature, 2901 ± 10 K). The radius of the spectral measurement was 10 mm. Spectral reflectance patterns of the first fully developed leaf of 5‐wk‐old *J. vulgaris* seedlings were measured with the black panel face of the probe as background. The calibration was made using the white reference face of the leaf‐clip to validate the measurements (Carvalho *et al*., 2012). All measurements were corrected and the noisy region between 350 and 400 nm was eliminated using the software ViewSpec Pro (v.5.6.10; ASD Inc., Boulder, CO, USA). A number of hyperspectral indices were calculated to assess the responses of the plant spectral reflectance to shifts in soil community (see Table 1).

**Table 1:** Spectral indices used in this study to evaluate the spectral reflectance of *Jacobaea vulgaris* in response to soil conditioning and sieving

Abbreviation	Name	Description	References
REP	Red‐edge position	Chlorophyll content	Clevers *et al*. (2002)
mREP	Modified red‐edge position	Pigment content	Sims & Gamon (2002)
PRIb	Photosynthetic radiation index	Plant photosynthetic efficiency	Gamon *et al*. (1992)
NRI	Nitrogen reflection index	Related to nitrogen content	Filella *et al*. (1995)
PPR	Plant pigment ratio	Plant pigment stress	Metternicht (2003)
PSa	Plant stress index	General plant stress	Carter (1994)

### Chemical analysis

Each freeze‐dried leaf was ground to a fine homogeneous powder using a grinder (TissueLyser II, Qiagen, Germany) at 1800 rpm for 2 min. Five milligrams of leaf powder were suspended in 0.5 ml of 2% formic acid solution containing internal standard (heliotrine, 1 μg ml^−1^). After centrifugation and filtration, 25 μl of the aliquot was diluted with 975 μl of 10 mM ammonium hydroxide solution; 10 μl of diluted aliquot was injected into a liquid chromatography‐tandem mass spectrometer (LC‐MS/MS) composed of a Waters Acquity UPLC system (Waters, Milford, MA, USA) coupled to a Waters Premier XE tandem mass spectrometer (Waters). The mass spectrometer was operated in positive electrospray mode and the samples were screened for a total of 45 PAs with output data processed using Masslynx 4.1 software (Waters). Detailed information on the LC‐MS/MS procedure is described in Cheng *et al*. (2011).

### Molecular analyses of the bacterial and fungal community composition in the inocula

The stored samples of watery inocula (2 ml per sample of each of the 1000‐, 20‐, 5‐ and 0.2‐μm inocula) were freeze‐dried under vacuum (−55°C, Labconco Free Zone 12 L Freeze Dry System). DNA was extracted from freeze‐dried samples of each watery inoculum (*n* = 5) and from 0.25 g of soil (‘whole soil’ treatment, *n* = 5) using a PowerSoil DNA Isolation Kit (Mo Bio Laboratories, Carlsbad, CA, USA) following the manufacturer's protocol. The DNA quantity was measured using a Nanodrop spectrophotometer (Thermo Scientific, Hudson, NH, USA). Between 5 and 10 ng of DNA was employed for PCR using the primers ITS4ngs and ITS3mix targeting the ITS2 region of fungal genes (Tedersoo *et al*., 2015). For bacteria, the primers 515FB and 806RB (Caporaso *et al*., 2012), targeting the V4 region of the 16Sr RNA gene, were used. Phusion Flash High‐Fidelity PCR Master Mix (Thermo Scientific) was used according to the manufacturer's instructions. The cycling conditions for bacteria were 98°C for 3 min, followed by 25 or 30 cycles of 98°C for 45 s, 50°C for 60 s and 72°C for 90 s. The cycling conditions for fungi were 98°C for 3 min, followed by 30 or 35 cycles of 98°C for 45 s, 55°C for 60 s and 72°C for 90 s. Final extension for both was 72°C for 10 min. For fungi, initially 30 cycles of PCR were performed and, in the absence of a band, another reaction with 35 cycles was performed (Tedersoo *et al*., 2015). For bacteria, initial PCRs had 25 cycles and subsequent PCRs 30 cycles. Both positive control (DNA from pure cultures) and negative control (water) were used in the amplification. The presence of PCR product was checked using agarose gel electrophoresis. The PCR products were purified using Agencourt AMPure XP magnetic beads (Beckman Coulter, Brea, CA, USA). Adapters and barcodes were added to samples using a Nextera XT DNA library preparation kit set A (Illumina, San Diego, CA, USA). The final PCR product was purified again with AMPure beads, checked using agarose gel electrophoresis and quantified with a Nanodrop spectrophotometer before equimolar pooling. The average concentrations of DNA obtained from the 0.2‐ and 5.0‐μm mesh sizes were <2 and <3 ng μl^−1^, respectively. From the 0.2‐μm inoculum, amplification of bacterial DNA was only achieved for six of 20 samples (five replicates × four treatments), but from all samples of the other inocula DNA amplification was successful. No DNA amplification of fungi was achieved in any of the replicates of the 0.2‐ and 5‐μm inocula. Consequently, the final libraries of bacteria and fungi consisted of 86 and 60 samples, respectively.

Libraries were sequenced at BGI (Shenzhen, China) using MiSeq PE250 for bacteria and MiSeq PE300 for fungi (Illumina, San Diego, CA, USA). The data were analyzed using an in‐house pipeline (de Hollander, 2017). Reads were first quality filtered (base quality value > 25, read length between 100 bp and 700 bp with at least 20‐bp overlap). For fungi, only those reads mapped to the internal transcribed spacer (ITS) region were included for analysis. In total, an averaged number of 84 101 and 54 966 reads per sample for bacteria and fungi, respectively, were obtained. The SILVA database was used to classify bacteria and the UNITE database (Abarenkov *et al*., 2010) was used for the identification of fungi. The ITSx extractor was used to identify fungal ITS regions (Nilsson *et al*., 2010). FUNGuild (Nguyen *et al*., 2016) was used to classify fungal operational taxonomic units (OTUs) into potential functions. The OTUs that could be classified were grouped into saprophytes, arbuscular mycorrhizal fungi (AMFs), plant pathogens, plant endophytes and the rest (fungal/animal/unidentified plant pathogens).

### Data analysis

All data on biomass, PA concentrations and spectral indices in the plant foliage were analyzed using ANOVA with plant conditioning (NP, P, P+B and P+A) and type of watery inoculum (1000, 20, 5, 0.2 μm) as fixed factors. Tukey's *post hoc* tests were used for multiple comparisons. Data from the four treatments (NP, P, P+B, P+A) for plants growing in pots with whole soil inoculum were analyzed separately using one‐way ANOVA. To compare the sterile soil treatment with each watery inoculum treatment, the above‐mentioned data and data from the sterile treatment were analyzed using one‐way ANOVA with all soil inocula included as factor, regardless of the type of plant conditioning or type of soil inoculum, followed by a Dunnett *post hoc* test. We used principal component analysis (PCA) and redundancy analyses (RDAs) to examine the influence of the soil inocula and soil conditioning on the composition of foliar PAs. Significances in multivariate analysis were tested using a Monte Carlo permutation test (999 permutations).

To meet the assumptions of ANOVA tests, data were checked for homogeneity of variance using a Levene test and for normality using a Kolmogorov–Smirnov test. The data on root biomass in the one‐way ANOVA were log_10_‐transformed and the data on total PA concentration and proportion of tertiary PAs were square‐root transformed. Multivariate analyses were performed using canoco, v.5.03 (Šmilauer & Lepš, 2014) and all other analyses were carried out in R v.3.2.5 (R Core Team, 2016).

All samples in which bacterial or fungal DNA was detected were included in the multivariate analyses of bacterial and fungal composition. The influence of the number of reads per sample was tested by adding it as a variable, but it did not significantly explain the community structure of fungi or bacteria (PERMANOVA for both, *P* > 0.05). Data of sequenced libraries in the inocula were normalized using total sum scaling (TSS). Effects of conditioning and inoculum treatments on the structure of the bacterial and fungal community were then examined using PERMANOVA with Bray–Curtis dissimilarity in R (package vegan). Separations among treatments were visualized using nonmetric multidimensional scaling (NMDS) of a Bray–Curtis dissimilarity matrix.

## Results

### Plant growth and defense in soil with ‘whole soil’ inocula

**Figure 2:**
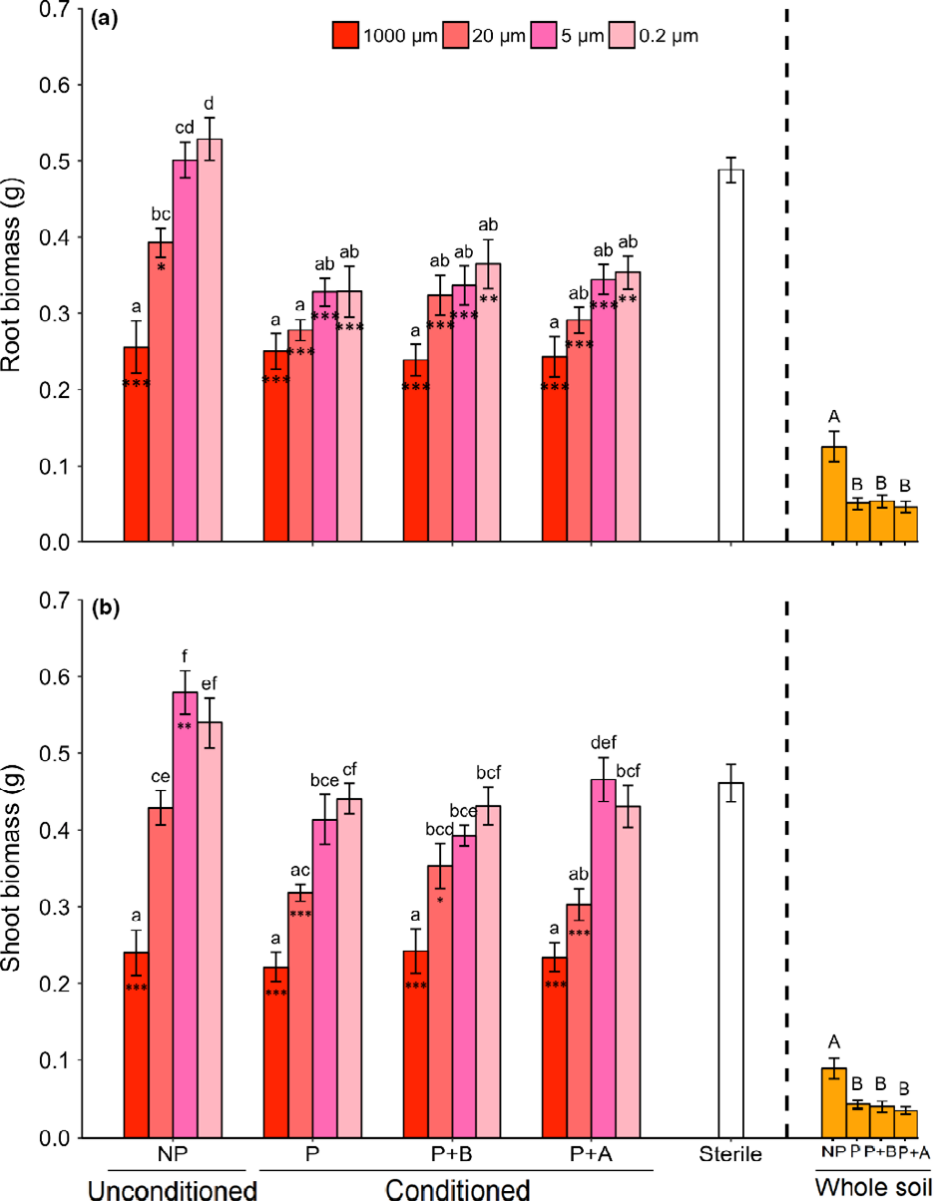
Mean (± SE) (a) root biomass and (b) shoot biomass of *Jacobaea vulgaris* plants growing in pots with sterilized soil and soil inocula created from unconditioned soil (NP), soil conditioned by plants (P), soil conditioned by plants exposed to belowground herbivory (P+B) or soil conditioned by plants exposed to aboveground herbivory (P+A). Soil suspension was passed through different meshes to obtain 1000‐, 20‐, 5‐ and 0.2‐μm inocula. The ‘Sterile’ treatment consisted of sterilized soil and demineralized water. Bars with identical letters are not significantly different based on a Tukey *post hoc* test; asterisks within each bar denote significant differences from the sterile treatment based on Dunnett's test: *, *P *<* *0.05; **, *P *<* *0.01; ***, *P *<* *0.001. The ‘Whole soil’ treatments consisted of sterilized soil mixed with whole field soil (1 : 1, w/w) created from the NP, P, P+B and P+A conditioning treatments. Bars with identical capital letters are not significantly different at *P *=* *0.05 among the ‘Whole soil’ treatments according to one‐way ANOVA.

**Figure 3:**
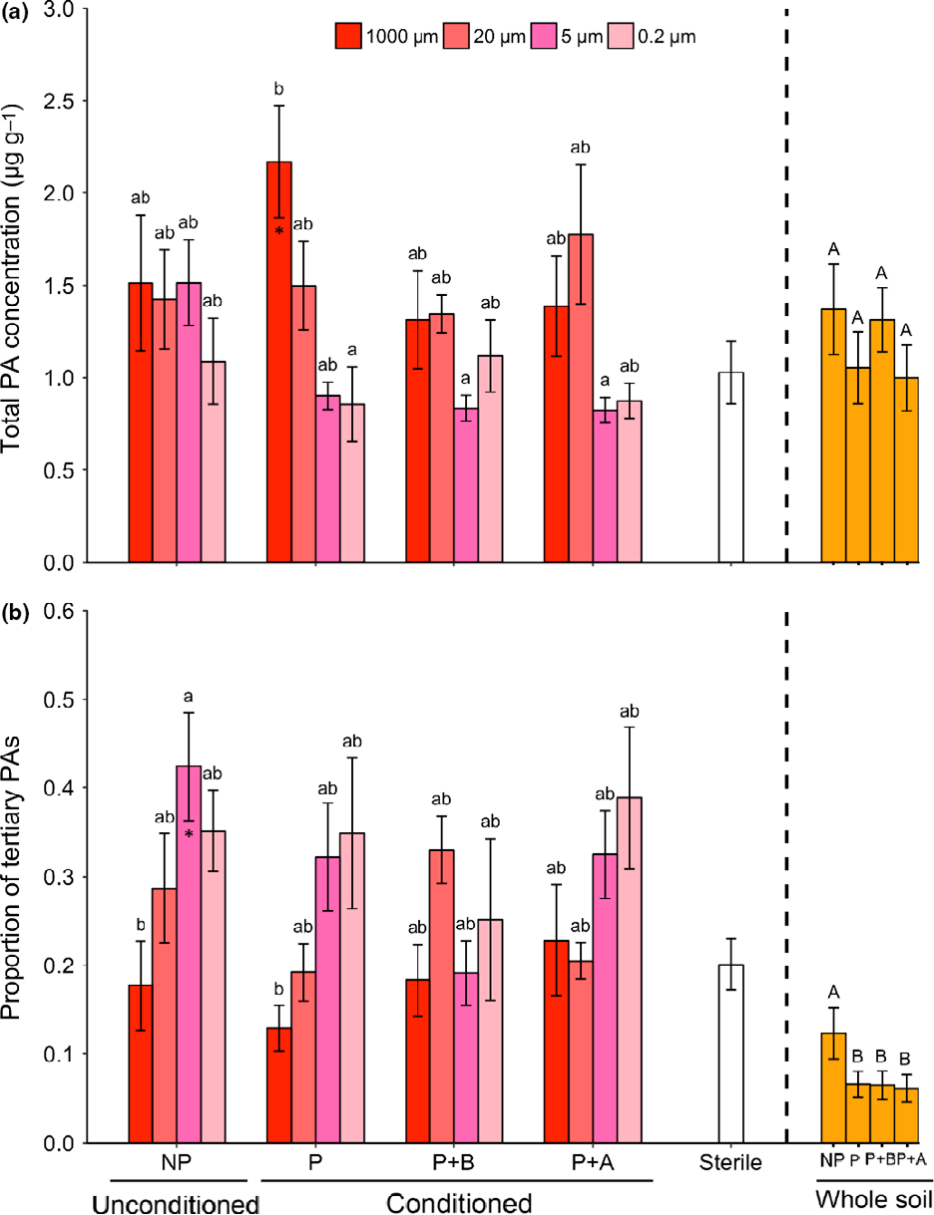
Mean (± SE) (a) total pyrrolizidine alkaloid (PA) concentration, and (b) proportion of tertiary PAs in the foliage of *Jacobaea vulgaris* plants growing in pots with sterilized soil and soil inocula created from unconditioned soil (NP), soil conditioned by plants (P), soil conditioned by plants exposed to belowground herbivory (P+B) or soil conditioned by plants exposed to aboveground herbivory (P+A). Soil suspension was passed through different meshes to obtain 1000‐, 20‐, 5‐ and 0.2‐μm inocula. The ‘Sterile’ treatment consisted of sterilized soil and demineralized water. Bars with identical letters are not significantly different based on a Tukey *post hoc* test; asterisks within each bar denote significant differences from the sterile treatment based on Dunnett's test: *, *P *<* *0.05. ‘Whole soil’ treatments consisted of sterilized soil mixed with whole field soil (1 : 1, w/w) created from the NP, P, P+B and P+A conditioning treatments. Bars with identical capital letters are not significantly different at *P *<* *0.05 among the ‘Whole soil’ treatments according to one‐way ANOVA.

**Figure 4:**
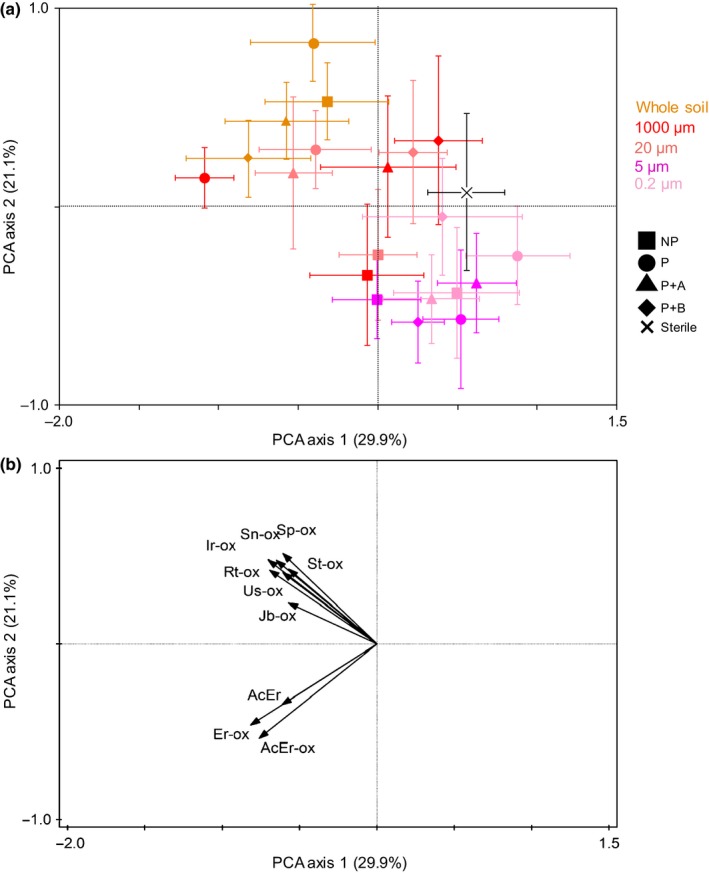
Ordination diagram of a principal component analysis (PCA) of the shoot pyrrolizidine alkaloid (PA) composition of *Jacobaea vulgaris* (a). The mean (± SE) sample scores of plants grown in sterilized soil mixed with whole soil, or inoculated with watery soil extracts from unconditioned soil (NP, squares), soil conditioned by undamaged plants (P, circles) and soil conditioned by plants exposed to aboveground (P+A, triangles) or belowground (P+B, diamonds) herbivores are shown; all PAs with > 50% fit are shown in (b). The ‘Sterile’ treatment (black cross) consisted of sterilized soil and demineralized water. The color of each point corresponds to the inoculum treatment. Percentages of total explained variation by PCA axes are given in parentheses. AcEr, acetylerucifoline; Er, erucifoline; Ir, integerrimine; Jb, jacobine; Rt, retrorsine; Sn, senecionine; Sp, seneciphylline; St, spartioidine; Us, usaramine; ‐ox is the N‐oxide form of the corresponding PA.

*Jacobaea vulgaris* plants growing in pots with whole soil added produced, overall, less plant biomass than plants growing in sterilized soil. Further, plants grown in pots inoculated with conditioned whole soil produced less biomass than plants growing in pots with unconditioned whole soil, independent of plant exposure to AG or BG herbivores during the conditioning phase (Fig. 2a,b). Total PA concentration in the foliage of plants growing in pots in which whole soil was added did not differ from that of plants growing in sterilized soil (Fig. 3a). However, overall, plants growing in pots with whole soil had a lower proportion of tertiary PAs than plants growing in sterilized soil, irrespective of the conditioning treatment from which the whole soil was created. The proportion of tertiary PAs of plants grown in pots in which conditioned soil was added was lower than that of plants grown in pots with unconditioned soil (Fig. 3b). The PA composition of plants growing in pots in which whole soil was added differed from the PA composition of plants growing in pots with sterilized soil, regardless of the conditioning treatments of the whole soil (Fig. 4a).

### Plant growth and defense in soil with ‘watery inocula’

Plant biomass was lower in pots with conditioned than in pots with unconditioned watery inocula, and this was independent of plant exposure to AG or BG herbivores during the conditioning phase (Table 2; Fig. 2). This effect was only significant for inocula sieved through mesh sizes smaller than 1000 μm, and plant biomass tended to increase with decreasing mesh sizes (Fig. 2). Root biomass was lower in pots inoculated with 1000‐μm inoculum than in pots with other watery inocula (20, 5 and 0.2 μm), but this was only significant when these inocula were created from unconditioned soil (Table 2; Fig. 2a). The shoot biomass of plants in soil with 1000‐ or 20‐μm watery inocula was lower than that in soil with 5‐ or 0.2‐μm inocula, and this effect was independent of whether the inocula were created from soil conditioned by a plant, and whether the plant was exposed to AG or BG herbivores (Table 2; Fig. 2b).

**Table 2:** ANOVA results for effects of soil conditioning (NP, P, P+B and P+A) and soil inoculum (1000, 20, 5 and 0.2 μm) on plant growth and the concentration of pyrrolizidine alkaloids (PAs) in the foliage of *Jacobaea vulgaris* plants

Treatment		Plant growth				PAs			
		Shoot biomass (g)		Root biomass (g)		Total PA (mg g^−1^ DW)		Proportion of tertiary PAs	
	df	*F*	*P*	*F*	*P*	*F*	*P*	*F*	*P*
Conditioning^a^	3	10.03	**<0.001** c	20.34	**<0.001**	0.65	0.583	1.55	0.206
Inoculum^b^	3	77.07	**<0.001**	30.11	**<0.001**	5.87	**<0.001**	8.14	**<0.001**
Conditioning × inoculum	9	1.91	0.056	2.79	**0.005**	1.67	0.102	1.81	0.073
Error	132								

a Conditioning treatments include soil that was not conditioned (NP), soil conditioned by undamaged *J. vulgaris* plants (P), or by *J. vulgaris* plants that were exposed to belowground (P+B) or aboveground (P+A) insect herbivores.

b Inocula include soil suspensions that were sieved through 1000‐, 20‐, 5‐ or 0.2‐μm mesh sizes.

c Bold *P* values indicate significant effects at *P *<* *0.05.

In comparison with plants growing in sterilized soil, *J. vulgaris* plants produced less root biomass in pots with watery inocula extracted from conditioned soil, irrespective of the sieving level, and in pots with unconditioned inocula sieved through 1000‐ and 20‐μm meshes (Fig. 2a). Shoot biomass was lower for plants growing in pots with 1000‐μm inoculum than for plants growing in sterilized soil, regardless of whether this inoculum originated from conditioned or unconditioned soil (Fig. 2b). Shoot biomass was also lower for plants in pots with 20‐μm inoculum, but only when the inoculum was created from plant‐conditioned soils (Fig. 2b). In contrast, shoot biomass in pots inoculated with 5‐μm inoculum was higher than in pots with sterilized soil, but only when the inoculum was created from unconditioned soil (Fig. 2b).

For plants that received watery inocula, the total PA concentration in the foliage of *J. vulgaris* was not influenced by soil conditioning, but differed significantly between the sieving treatments. Plants had higher total PA concentrations when grown in pots with 1000‐ and 20‐μm inocula than when grown with 5‐ and 0.2‐μm inocula, regardless of the conditioning of the soil from which the inocula were created (Table 2; Fig. 3a). Leaves of *J. vulgaris* plants growing in pots with 1000‐μm inoculum also had higher total PA concentrations than leaves of plants grown in sterilized soil, but only when the inoculum was created from soil in which previously an undamaged plant had been grown (Dunnett test: *P* = 0.023; Fig. 3a). The proportion of tertiary PAs in pots with watery inocula generally increased with decreasing mesh size, up to 5 μm (Table 2; Fig. 3b). At the smallest mesh sizes (5‐ and 0.2‐μm inocula), the proportion of tertiary PAs tended to be higher for plants growing in pots with watery inocula than for plants growing in 100% sterilized soil (Fig. 3b).

Thirty‐five different PAs were detected in the leaves of *J. vulgaris* plants. The first two PCA axes explained 51% of the variation in PA composition (Fig. 4a). The PA composition was significantly affected by the interactive effect of soil conditioning and soil inocula (RDA: *F* = 2.5, *P* = 0.001, 22% explained variation). The PA composition of plants growing in pots in which whole soil was added was not different from that of plants grown in pots in which 1000‐ and 20‐μm inocula were added, but differed from plants growing with watery inocula created using smaller mesh sizes (5 and 0.2 μm). Erucifoline‐type PAs contributed most to the separation between plants grown with watery inocula created from unconditioned soil and plants grown with watery inocula created from soil in which plants damaged by BG herbivores had been grown, but only when these inocula had been sieved through larger mesh sizes (Fig. 4b). Senecionine‐ and jacobine‐type PAs contributed most to the separation of plants grown in pots with watery inocula sieved through meshes larger than 20 μm from plants in pots with watery inocula sieved through smaller sized meshes, but this separation occurred only when these inocula originated from soil conditioned by undamaged plants or by plants damaged by AG herbivores (Fig. 4b).

### Spectral indices of plants growing with all soil inocula

**Figure 5:**
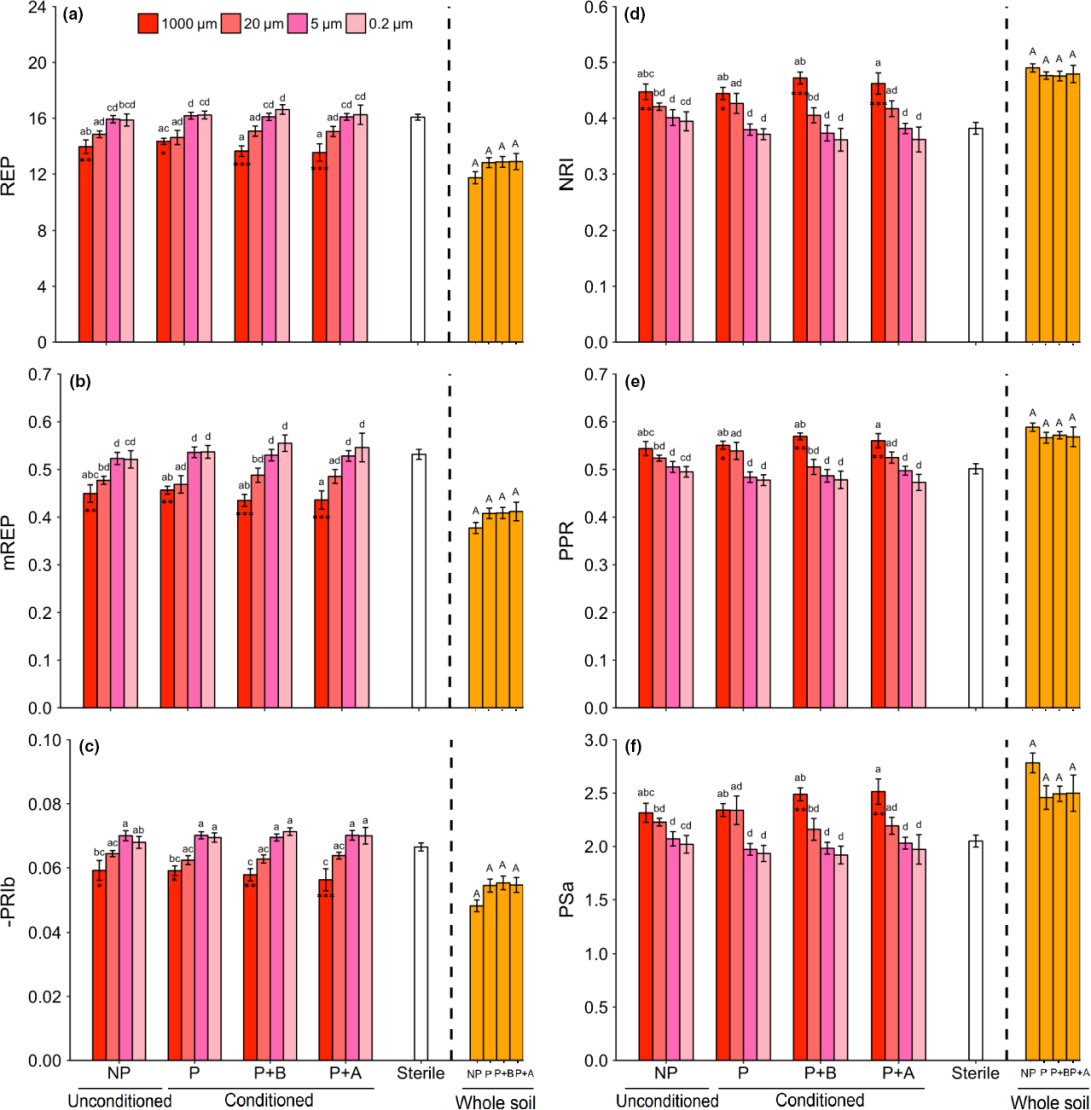
Spectral indices representing plant (a–c) quality and (d–f) stress in *Jacobaea vulgaris* species growing in pots with soil inocula (1000, 20, 5 and 0.2 μm) created from unconditioned soil (NP), from soil in which an undamaged plant had been grown (P) or from soil in which a plant exposed to belowground (P+B) or aboveground (P+A) herbivores had been grown. Soil suspension was passed through different meshes to obtain 1000‐, 20‐, 5‐ and 0.2‐μm inocula. The ‘Sterile’ treatment consisted of sterilized soil and demineralized water. Bars with identical letters are not significantly different based on Tukey's *post hoc* test; asterisks within each bar denote significant differences from the sterile treatment based on Dunnett's test: *, *P *<* *0.05; **, *P *<* *0.01; ***, *P *<* *0.001. The spectral indices are explained in Table 1. The ‘Whole soil’ treatments consisted of sterilized soil mixed with whole soil (1 : 1, w/w) created from NP, P, P+B and P+A conditioning treatments. Bars with identical capital letters are not significantly different at the *P *<* *0.05 level among the ‘Whole soil’ treatments according to one‐way ANOVA. REP, red‐edge position; mREP, modified red‐edge position; PRIb, photosynthetic radiation index; NRI, nitrogen reflection index; PPR, plant pigment ratio; PSa, plant stress index.

Overall, spectral indices were unaffected by whether a plant had been grown in the soil used to create the inocula, and whether the plant was exposed to herbivory, but there was a strong effect of mesh size for plants growing in pots with watery inocula (Table 3; Fig. 5). Depending on the index, the values increased or decreased with decreasing mesh size. *Jacobaea vulgaris* plants growing in pots in which whole soil was added and in pots with 1000‐μm inoculum had lower red‐edge position (REP), modified red‐edge position (mREP) and photosynthetic radiation index (PRIb) than plants growing in sterilized soil (Fig. 5a–c), whereas the nitrogen reflection index (NRI), plant pigment ratio (PPR) and plant stress index (PSa) tended to be higher for plants grown in these soils (Fig. 5d–f).

**Table 3:** ANOVA results for the effects of soil conditioning (NP, P, P+B and P+A) and soil inoculum (1000, 20, 5 and 0.2 μm) on the photospectral indices of leaves of *Jacobaea vulgaris* plants

Treatment		REP		mREP		PRIb		NRI		PPR		PSa	
	df	*F*	*P*	*F*	*P*	*F*	*P*	*F*	*P*	*F*	*P*	*F*	*P*
Conditioning^a^	3	1.60	0.193	0.14	0.934	0.13	0.945	0.40	0.751	0.10	0.958	0.16	0.925
Inoculum^b^	3	7.96	**<0.001** c	34.27	**<0.001**	37.78	**<0.001**	29.98	**<0.001**	28.34	**<0.001**	31.34	**<0.001**
Conditioning × inoculum	9	1.84	0.066	0.50	0.871	0.41	0.927	0.85	0.570	1.01	0.435	0.42	0.921
Error	124												

REP, red‐edge position; mREP, modified red‐edge position; PRIb, photosynthetic radiation index; NRI, nitrogen reflection index; PPR, plant pigment ratio; PSa, plant stress index.

a Conditioning treatments include soil that was not conditioned (NP), soil conditioned by undamaged *J. vulgaris* plants (P), or by *J. vulgaris* plants that were exposed to belowground (P+B) or aboveground (P+A) insect herbivory.

b Inocula include soil suspensions that were sieved through 1000‐, 20‐, 5‐ or 0.2‐μm mesh sizes.

c Bold *P* values indicate significant effects at *P *<* *0.05.

### Microbial community structure in all soil inocula

**Figure 6:**
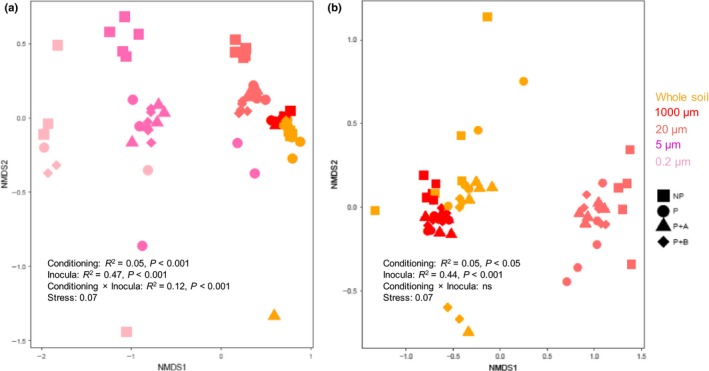
Nonmetric multidimensional scaling (NMDS) of (a) bacterial and (b) fungal community composition in watery inocula (1000, 20, 5 and 0.2 μm) and ‘Whole soil’ inocula created from unconditioned soil (NP, squares), from soil in which an undamaged *Jacobaea vulgaris* plant had been grown (P, circles) or from soil in which a plant exposed to aboveground (P+A, triangles) or belowground (P+B, diamonds) herbivores had been grown. Colors correspond to different inoculum treatments. Effects of conditioning and soil inocula (mesh size) on the community compositions were assessed using PERMANOVA. *R* ^2^ values represent the proportional variations of bacterial or fungal community composition explained by conditioning or inocula. Stress values are included as a measure of ‘goodness of fit’ for the NMDS.

Only samples with amplified DNA detected were included in the analysis. The type of inoculum (watery inocula and whole soil inoculum) strongly influenced the community structure of both fungi and bacteria, with over 40% of the variation explained (Fig. 6a,b). For both bacteria and fungi, the community compositions in whole soil and in 1000‐μm watery inoculum were similar, and differed from the watery inocula sieved through smaller mesh sizes. Soil conditioning explained less variation (*c*. 5%) than the type of inoculum, but also significantly affected bacterial and fungal community composition. Soil conditioning treatments (i.e. whether a plant had been grown in the soil and whether the plant was exposed to herbivory) significantly influenced bacterial community composition in whole soil, and in the 1000‐, 20‐ and 5‐μm inocula, but not in the 0.2‐μm inoculum, resulting in a significant interaction effect with soil inocula (*R* ^2^ = 0.12, *P *<* *0.001, Fig. 6a). Soil conditioning influenced fungal community composition in all inocula in which fungi were present (whole soil, 1000‐ and 20‐μm watery inocula; *R* ^2^ = 0.05, *P *<* *0.001, Fig. 6b). The strongest effects of soil conditioning on both bacterial and fungal communities occurred in the 20‐μm inoculum, with 59% and 35%, respectively, of the variation explained (Supporting information Table S1).

## Discussion

Our study shows that selective removal of biota from soil increases, overall, the growth of *J. vulgaris* and influences plant defense responses. *Jacobaea vulgaris* plants had higher total PA concentrations in the foliage when grown in pots with both microbial and mesofaunal groups (> 20 μm), and the removal of mesofaunal groups resulted in a lower concentration of N‐oxide PAs as well as higher proportions of tertiary alkaloids, the more toxic form of PAs. Interestingly, the PA composition of plants growing in soil with watery inocula sieved through larger mesh sizes (1000 and 20 μm) differed from that of plants grown in soil with watery inocula sieved through smaller mesh sizes (5 and 0.2 μm) in which soil fungi were excluded or at least greatly reduced. However, this difference only occurred when the watery inocula were obtained from soil in which, previously, either an undamaged plant or a plant damaged by AG herbivores had been grown. Selective removal of groups of soil organisms also led to changes in hyperspectral reflectance patterns, indicating that the presence of different groups of soil organisms also changed the overall chemical composition of the plant. Whole soil inoculum and 1000‐μm watery inoculum had similar bacterial and fungal communities, but these differed from those of the smaller sized inocula. Soil conditioning effects within each inoculum type primarily occurred between unconditioned soil and the plant‐conditioned soils, representing strong effects of both soil group removals and PSFs.

Ragwort is a plant species that responds negatively to soil conditioning by the same species, but also by many other species (van de Voorde *et al*., 2011; Kos *et al*., 2015). Our results confirm this finding: adding whole soil to sterilized soil considerably reduced plant growth, independent of whether or not ragwort had been grown in the soil (Fig. 2). Importantly, although there had been no plant growing in the pots with ‘unconditioned’ soil during the conditioning phase, this soil was already conditioned because it had been collected from a grassland in which plants were growing at the time of collection. When this whole soil was dissected by a sieving approach in our study, we found that the negative feedback effect on shoot biomass was only apparent when the larger sized soil organisms were present in the inoculum (Fig. 2). The shoot biomass of plants in pots inoculated with the 0.2‐μm inoculum was even higher than that in sterilized soil. We expect that this may be caused by nutrients that are extracted from the donor soil in the water. By contrast, the negative feedback effect on *J. vulgaris* root biomass remained present even at this lowest mesh size, but only in soil in which the plant had been grown previously. All of these results together suggest that: soil organisms, in particular the larger sized microorganisms, such as fungi, which are already present in the soil when collected from the field, reduce plant growth; but also that there is a non‐microbial effect, and that, for example, plant exudates in the soil can be (partly) responsible for the observed negative feedback, as there was still a negative effect on root biomass at the smallest mesh size, when virtually all soil microorganisms were removed from the soil.

It is important to note that the addition of whole soil to sterilized soil exerted, overall, a stronger negative feedback effect on plant growth than the inoculation of watery inocula, even for the 1000‐μm inoculum which had a similar microbial community to the whole soil (Fig. 6). This may be a result of incomplete extraction and loss of viability of soil organisms caused by the filtering process (which can weaken the soil feedbacks on plant growth) or, alternatively, because these soil organisms could not exert their function (plant suppression) as efficiently in the watery inocula as in a soil microclimate (Mendes *et al*., 1999). Moreover, by adding the watery inocula we also inevitably added nutrients. This may also explain why the 1000‐μm inoculum with the most complete soil community resulted in a weaker negative PSF than observed in pots in which we added 50% whole soil. All of the above‐mentioned differences between the watery inocula and whole soil inoculum suggest that a direct comparison between these two types of inocula is inappropriate.

We hypothesized that insect herbivory would modify PSF effects as it can alter the rhizodeposition of the plant, which influences the soil microbial community or the concentration of plant‐exuded molecules in the soil, which, in turn, influence the performance of later growing plants (Kostenko *et al*., 2012; Bezemer *et al*., 2013). Kostenko *et al*. (2012) reported that the exposure of *J. vulgaris* to AG herbivory during the conditioning phase resulted in reduced root growth and lower PA concentrations of conspecific plants in the feedback phase. In our study, we did not find this PSF effect of herbivory on plant growth. Given that the seeds used in these two studies were collected from plants growing in different semi‐natural grasslands and in different years, the two experiments may have used different plant populations. Moreover, the microbial composition of the grassland soil that was used for the experiments may also have changed over time (Hannula *et al*., 2017; Morriën *et al*., 2017). Hence, the discrepancy between the experiments in soil feedback effects of herbivory may have been caused by intraspecific variation amongst *J. vulgaris* populations in the response to soil and to herbivory, or by differences in soil microbial composition (Schweitzer *et al*., 2008; Gottel *et al*., 2011; van de Voorde *et al*., 2012).

The PA composition of the plants in the feedback phase was affected by the soil conditioning treatments in a similar manner to that reported by Kostenko *et al*. (2012). For example, Kostenko *et al*. (2012) showed that the exposure of *J. vulgaris* plants to the BG herbivore *A. lineatus* during the conditioning phase led to changes in the PA composition of subsequently growing plants. In our study, the PA composition of *J. vulgaris* plants growing in the soil conditioned by plants exposed to this BG herbivore species also differed from that of the plants growing in soil conditioned by undamaged plants, by plants exposed to AG herbivory or in unconditioned soil. However, these differences disappeared when larger soil organisms, and especially fungi (>5 μm), were selectively removed from the soil (Figs 4a, 6b). This suggests that BG insect herbivory potentially modifies PSF effects on plant chemistry in the foliage of later growing plants via changes in soil fungi rather than bacteria. Given that the exposure of plants to BG herbivory did not affect the composition of the fungal community (Fig. 6b) or the relative abundances of plant‐associated fungi in the inocula (Table S2; Fig. S1), this modification may be achieved by changes in plant–fungus interactions during the response phase, which are caused by the inoculum rather than by changes in the fungal composition of the inoculum.

The selective removal of groups of soil organisms from the watery inocula also led to reduced concentrations of PAs in the leaves of *J. vulgaris*. Overall, PA concentrations were higher in plants exposed to watery inocula, which included all groups of soil organisms larger than 5 μm, than in plants with inocula in which the mesofauna was removed (Fig. 3a). Remarkably, plants in the treatments with the highest PA concentrations had the lowest biomass, in particular when growing in soil conditioned by undamaged plants. PAs are synthesized in the roots and the negative relationship between PA concentration and biomass suggests that plants that experienced more biotic stress invested relatively more in defense. Similarly, plants grown in pots with unconditioned field soil also had higher PA concentrations than in sterilized soil, although the biomass of these plants was lower. Clearly, these field soils may also contain microorganisms that induce defenses in plant foliage, particularly considering that these soils originated from a field with a history of high abundance of *J. vulgaris* (van de Voorde *et al*., 2011). The PA concentration in the foliage was higher, but the proportion of tertiary types of PA was lower, in plants growing in soil with 1000‐ or 20‐μm inocula than in soil with 5‐ or 0.2‐μm inocula. This suggests that fungi in the soil may be responsible for changes in PA concentrations and the shifts in PA forms in *J. vulgaris* plants. Several other studies have also indicated this, and have suggested that ragwort is sensitive to soil pathogenic fungi (Kostenko *et al*., 2012; Bezemer *et al*., 2013, 2018). Collectively, exposure of *J. vulgaris* plants to these fungi may reduce plant growth and increase total PA concentrations, as well as drive the transformation of PA forms.

The removal of groups of soil organisms correlated with the hyperspectral reflectance patterns of *J. vulgaris* leaves. This suggests that differences in soil communities changed the chemical composition of the plants, either directly by inducing plant defense responses or indirectly by influencing the growth and physiology of the plants (i.e. plant size or N content). Hyperspectral reflectance patterns in leaves have been used to detect the sensitivity of *J. vulgaris* to soils (Carvalho *et al*., 2016). Indices related to plant stress, such as PPR, NRI and PSa, were higher in plants exposed to whole soil or 1000‐ and 20‐μm watery inocula, in which larger sized soil organisms, such as fungi, were present, than in plants exposed to watery inocula without these organisms (Fig. 5). These results were consistent with those in a study showing that the exposure to soil fungi increases plant stress (Bezemer *et al*., 2013). Other indices, such as PRI and (m)REP, which are proxies of plant photosynthesis and nitrogen content, were lower in plants exposed to whole soil or 1000 and 20‐μm inocula. These results coincide with the overall higher total PA concentration in these plants and indirectly indicate higher plant stress levels.

In conclusion, this study shows that soil feedbacks mediated by a plant can affect the growth and defense of subsequently growing plants, but that these feedbacks can be strongly influenced by the selective removal of groups of soil biota in a size‐selective manner. Herbivory on the first plant did not alter the growth of a subsequent plant through soil feedbacks, but modulated these feedback effects on plant defensive chemistry. Results from our study also suggest that, in addition to the role of soil fungi in mediating plant growth and defense responses in *J. vulgaris* PSFs, non‐microbial effects, for example exudates secreted by the plant into the soil, can also contribute to these plant responses. Finally, the current study shows that PSF‐induced plant growth and defense responses can be related to changes in the hyperspectral reflectance patterns of plant leaves.

## Author contributions

WR and TMB planned the research. WR, MW and TMB performed the bioassay experiments, FB extracted the DNA, SEH prepared the sequencing library and analyzed the soil community, OK and PPJM measured PAs, and SC collected data on spectral reflectance. MW, WR, SEH, OK, WHvdP and TMB analyzed the data and wrote the manuscript with revisions from all authors.

## Supporting information

Please note: Wiley Blackwell are not responsible for the content or functionality of any Supporting Information supplied by the authors. Any queries (other than missing material) should be directed to the *New Phytologist* central office.

**Dataset S1** The dataset except sequence data used in this article.

**Fig. S1** Relative abundance of fungal groups.
**Table S1** Bacterial and fungal communities of each inoculum.
**Table S2** ANOVA results for the abundance of fungal groups.
