# Supplementary material for: Removal of soil biota alters soil feedback effects on plant growth and defense chemistry
Source: New Phytol. 2018 Oct 17;221(3):1478–91. doi: 10.1111/nph.15485 (PMC6587519; doi:10.1111/nph.15485)
Supplement: Supplementary file 2 — Fig. S1 Relative abundance of fungal groups. Table S1 Bacterial and fungal communities of each inoculum. Table S2 ANOVA results for the abundance of fungal groups. [file NPH-221-1478-s001.pdf]

Removal of soil biota alters soil feedback effects on plant growth and defense chemistry

Minggang Wang[1,2,3], Wei-bin Ruan [1,2*], Olga Kostenko[2], Sabrina Carvalho[2], S. Emilia Hannula[2], Patrick P.J. Mulder[4], Fengjiao Bu[5], Wim H. van der Putten[2,6], T. Martijn Bezemer[2,7]

[1]College of Life Sciences, Nankai University, Tianjin 300071, China
[2]Netherlands Institute of Ecology (NIOO-KNAW), Department of Terrestrial Ecology, PO Box 50, 6700 AB, Wageningen, The Netherlands
[3]Department of Plant Protection Biology, Swedish University of Agricultural Sciences, PO Box 102, SE-23053 Alnarp, Sweden
[4]RIKILT - Wageningen University & Research, PO Box 230, 6700 AE, Wageningen, The Netherlands
[5]Laboratory of Molecular Biology, Department of Plant Sciences, Wageningen University & Research, Wageningen, Netherlands
[6]Laboratory of Nematology, Wageningen University & Research, PO Box 8123, 6700 ES, Wageningen, The Netherlands
[7]Institute of Biology, Section Plant Ecology and Phytochemistry, Leiden University, PO Box 9505, 2300 RA Leiden, The Netherlands

*Corresponding author

Wei-bin Ruan
Address: College of Life Sciences, Nankai University, Tianjing 300071, China
Tel: 86-22-2350-4397
Fax: 86-22-2350-8800
Emails for correspondence: ruanweibin2004@hotmail.com

Supporting information

Table S1 PERMANOVA results for effects of soil conditioning[#] (NP, P, P+B and P+A) on the composition of bacterial and fungal communities in each of watery inocula (1000μm, 20μm, 5μm and 0.2μm) and in the whole soil inocula.

Inocula[a]	Bacterial community			Fungal community		
	$R^{2\,(b)}$	P	Stress[c]	R^2	P	Stress
Whole soil	0.26	< 0.05	0.07	0.25	< 0.05	0.07
1000μm	0.38	< 0.05	0.05	0.31	< 0.005	0.12
20μm	0.59	< 0.001	0.06	0.35	< 0.001	0.12
5μm	0.47	< 0.001	0.07	-	-	-
0.2μm	-	-	-	-	-	-

[#]Soil conditioning treatments includes soil that was not conditioned (NP), soil conditioned by *J. vulgaris* plants (P), or by *J. vulgaris* plants that were exposed to belowground (P+B) or aboveground (P+A) herbivores.

[a]Inocula indicates the soil suspensions that went through 1000 μm, 20 μm, 5 μm, 0.2 μm mesh size as well as the whole soil inocula, respectively.

[b]R^2 values represent the proportional variations of bacterial or fungal community composition explained by conditioning treatments.

[c]Stress values represent a measure of "goodness-of-fit" for the NMDS.

Table S2 ANOVA results for effects of soil conditioning (NP, P, P+B and P+A) and soil inocula (Whole soil, 1000µm, 20µm) on the relative abundances of different plant-associated fungi, including plant pathogens, arbuscular mycorrhizal fungi (AMF) and plant endophytes.

Treatment	*df*	Plant pathogenic fungi		AMF		Endophytes	
		F	P	F	P	F	P
Conditioning[a]	3	0.50	0.685	44.4	**<0.001**[c]	3.68	**0.018**
Inocula[b]	2	115.1	**<0.001**	17.3	**<0.001**	171.0	**<0.001**
Conditioning×Inocula	6	3.0	**0.014**	2.4	**0.041**	2.59	**0.030**
Error	48						

[a]Conditioning treatments includes soil that was not conditioned (NP), soil conditioned by undamaged *J. vulgaris* plants (P), or by *J. vulgaris* plants that were exposed to belowground (P+B) or aboveground (P+A) herbivores.

[b]Inocula indicates the soil suspensions that went through 1000µm, 20µm as well as the whole soil inocula.

[c]Bold p values indicate significant effects at P<0.05.

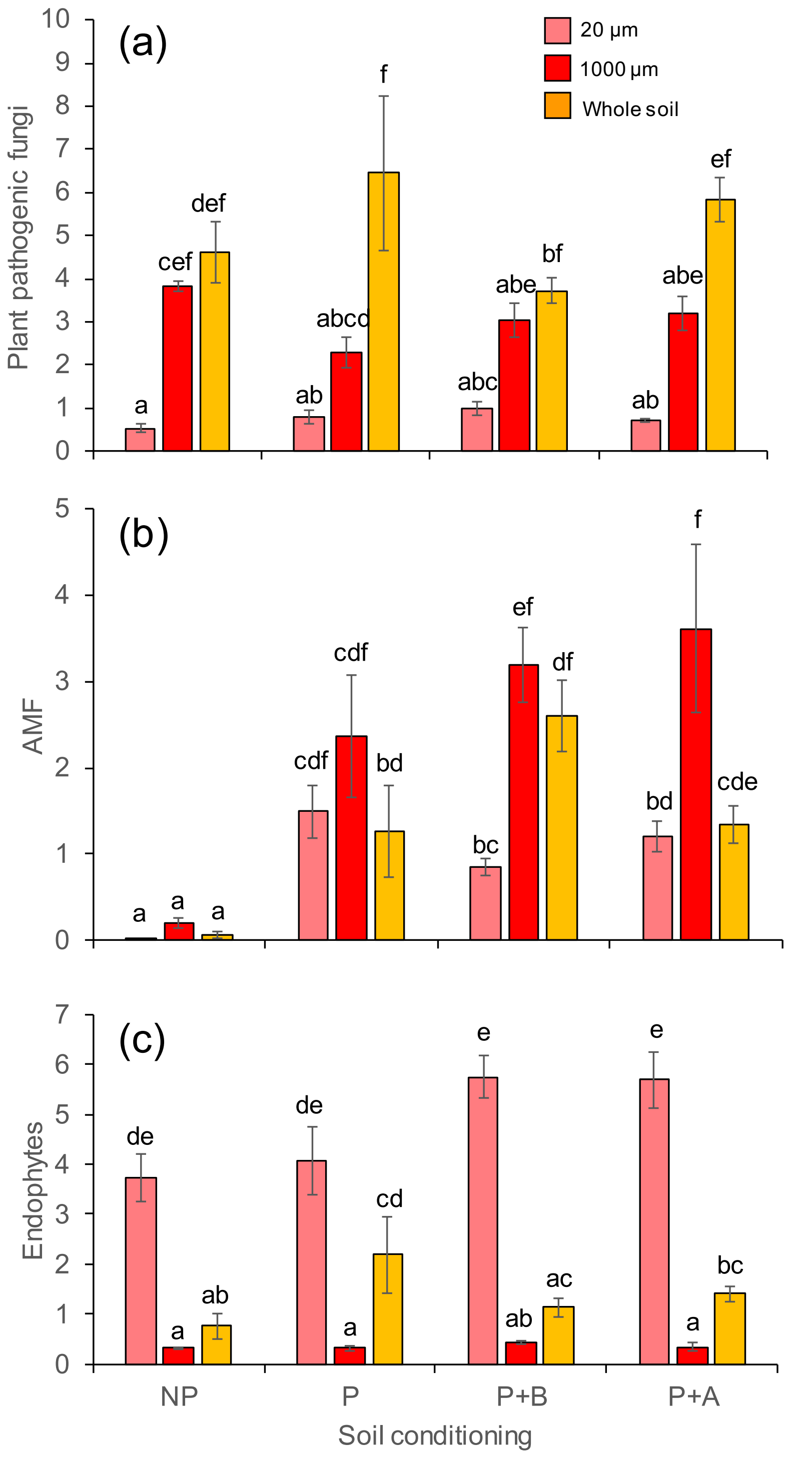

Fig. S1 Mean (± SE) relative abundances of plant pathogenic fungi (**a**), arbuscular mycorrhizal fungi (AMF, **b**) and plant endophytes (**c**) in 20μm, 1000μm and whole soil inocula that were created from unconditioned soil (NP), soil conditioned by plants (P), by plants exposed to belowground (P+B) or by plants exposed to aboveground (P+A) herbivory. Bars with identical letters are not significantly different based on a Tukey *post hoc* test at $p < 0.05$ level according to one-way ANOVA. Statistics are shown in Table S2.
